# Supplementary material for: Exploiting Gangliosides for the Therapy of Ewing’s Sarcoma and H3K27M-Mutant Diffuse Midline Glioma
Source: Cancers (Basel). 2021 Jan 29;13(3):520. doi: 10.3390/cancers13030520 (PMC7866294; doi:10.3390/cancers13030520)
Supplement: Supplementary file 1 [file cancers-13-00520-s001.zip › cancers-1068769-sup/Supplemental Figure S1.pdf]

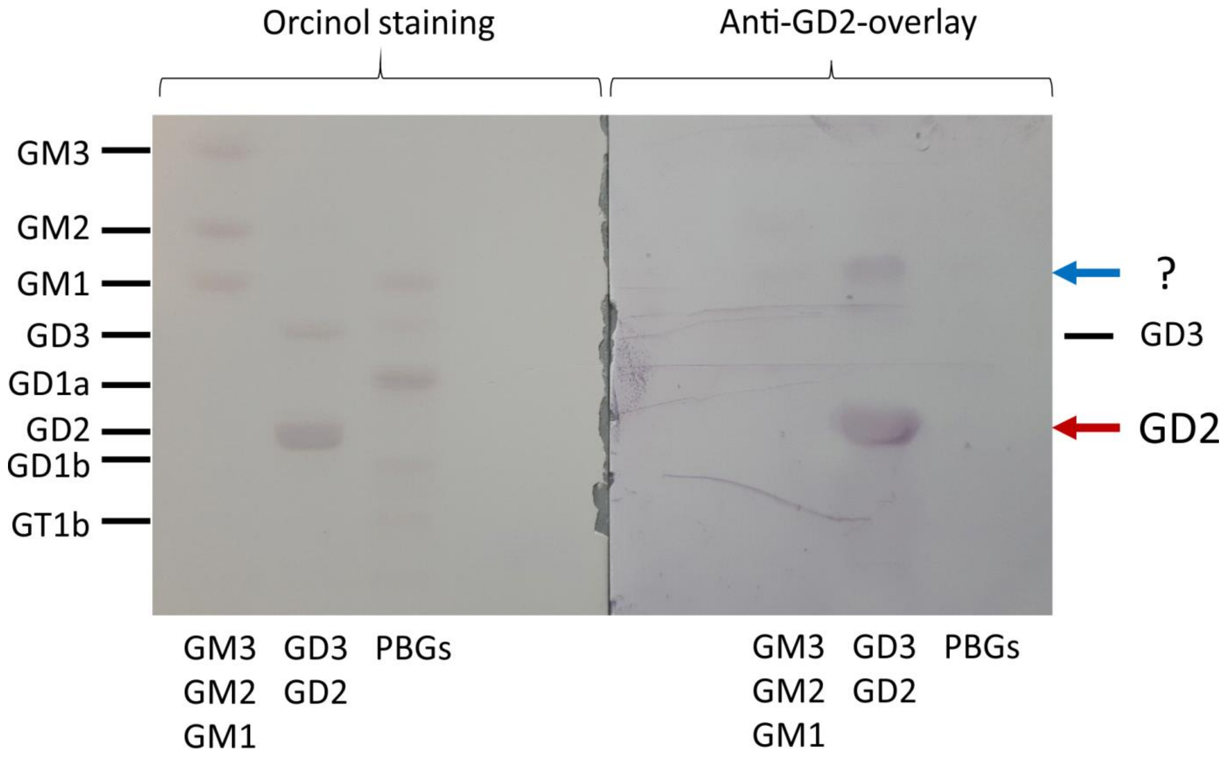

Supplemental Figure S1. Validation of the anti- GD2 antibody used for flow cytometry. From the gangliosides plotted (see orcinol staining) only GD2 was recognized (besides of an unknown impurity obtained from the commercial GD2 standard, which is labelled with a question mark.). Neither the biosynthetic precursor of GD2, namely GD3, nor the related monosialylated GM2, nor the biosynthetic downstream product GD1a were recognized by the anti-GD2 antibody.
