# Supplementary material for: Exploiting Gangliosides for the Therapy of Ewing’s Sarcoma and H3K27M-Mutant Diffuse Midline Glioma
Source: Cancers (Basel). 2021 Jan 29;13(3):520. doi: 10.3390/cancers13030520 (PMC7866294; doi:10.3390/cancers13030520)
Supplement: Supplementary file 1 [file cancers-13-00520-s001.zip › cancers-1068769-sup/Supplemental Figure S2.pdf]

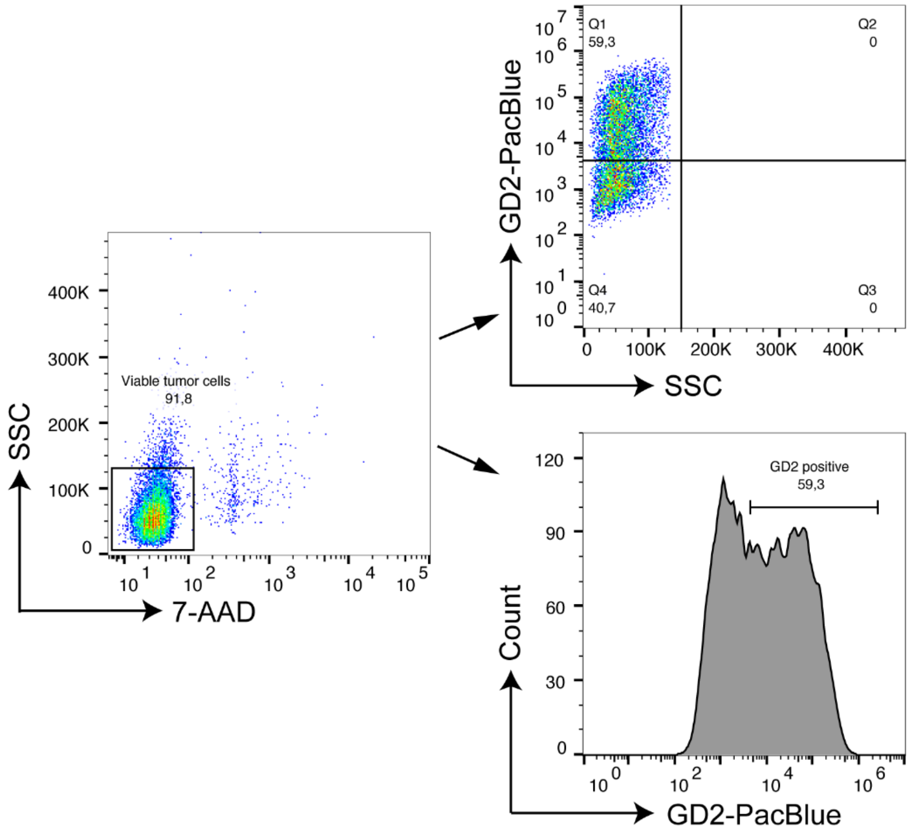

Supplemental Figure S2. Gating strategy

The 7-AAD against sideward scatter (SSC) plot was used to gate viable tumor cells. Dotplot with SSC against GD2 and GD2 histogram display the GD2 expression of viable tumor cells.
