# Supplementary material for: Exploiting Gangliosides for the Therapy of Ewing’s Sarcoma and H3K27M-Mutant Diffuse Midline Glioma
Source: Cancers (Basel). 2021 Jan 29;13(3):520. doi: 10.3390/cancers13030520 (PMC7866294; doi:10.3390/cancers13030520)
Supplement: Supplementary file 1 [file cancers-13-00520-s001.zip › cancers-1068769-sup/Supplemental Figure S3.pdf]

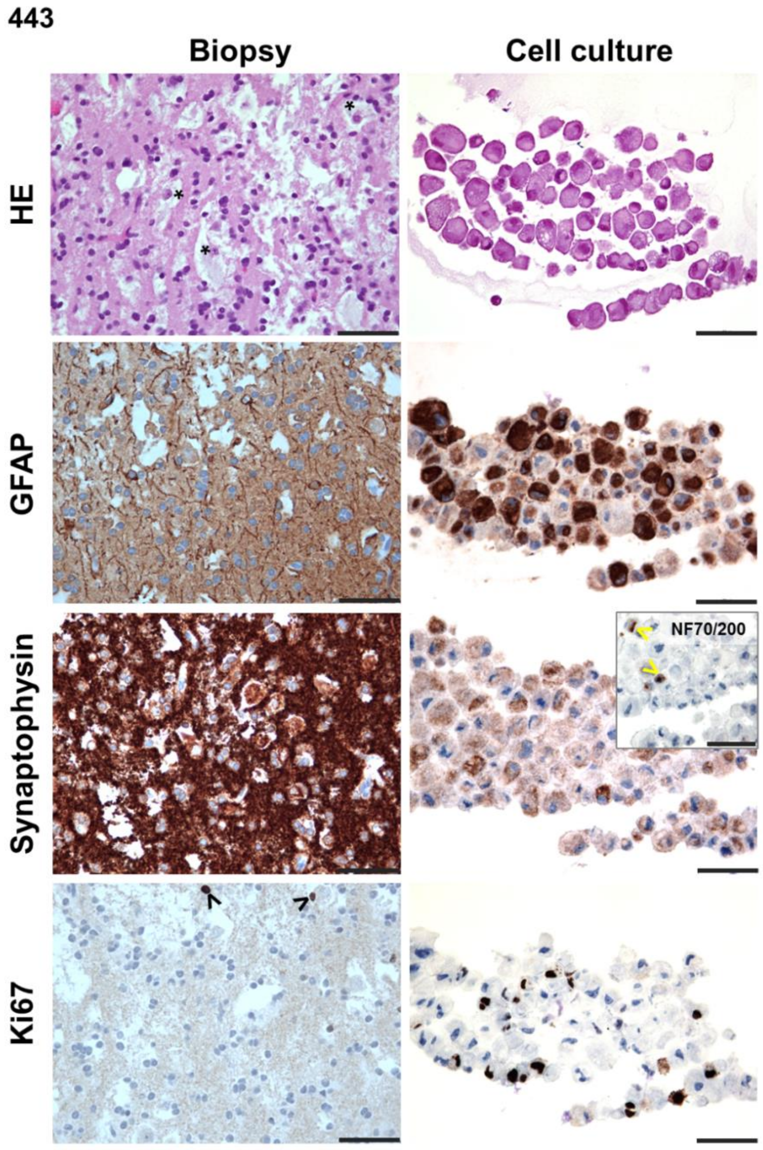

Supplemental Figure S3. Validation of DNET primary cells

Primary tumor cells of patient no 443 (dysembryoplastic neuroepithelial tumour = DNET) were embedded in paraffin, stained with HE and further analysed by IHC with the indicated antibodies. The tumor used for the isolation of the cell suspension was used as reference (biopsy, left panel). The tumor biopsy consisted of oligodendrocyte-like cells embedded in a mucoid matrix with so-called floating neurons (black asterisk). GFAP staining revealed GFAP-positive cells in the biopsy and in the cell culture. Further, few cultured cells showed immunoreactivity for Synaptophysin and, to a lesser extent, also for Neurofilament protein (NF70/200, yellow arrowhead) indicating the neuronal component of the DNET present in the cell culture. While proliferation in the biopsy was very low (Ki67, black arrowhead), the cultured cells showed a higher proliferation rate with many Ki67-labelled nuclei. However, this was not surprising as the cells were expanded before and processed for paraffin-embedding in the proliferative state. Scale bar 50 µm.
