# Supplementary material for: Exploiting Gangliosides for the Therapy of Ewing’s Sarcoma and H3K27M-Mutant Diffuse Midline Glioma
Source: Cancers (Basel). 2021 Jan 29;13(3):520. doi: 10.3390/cancers13030520 (PMC7866294; doi:10.3390/cancers13030520)
Supplement: Supplementary file 1 [file cancers-13-00520-s001.zip › cancers-1068769-sup/Supplemental Figure S4.pdf]

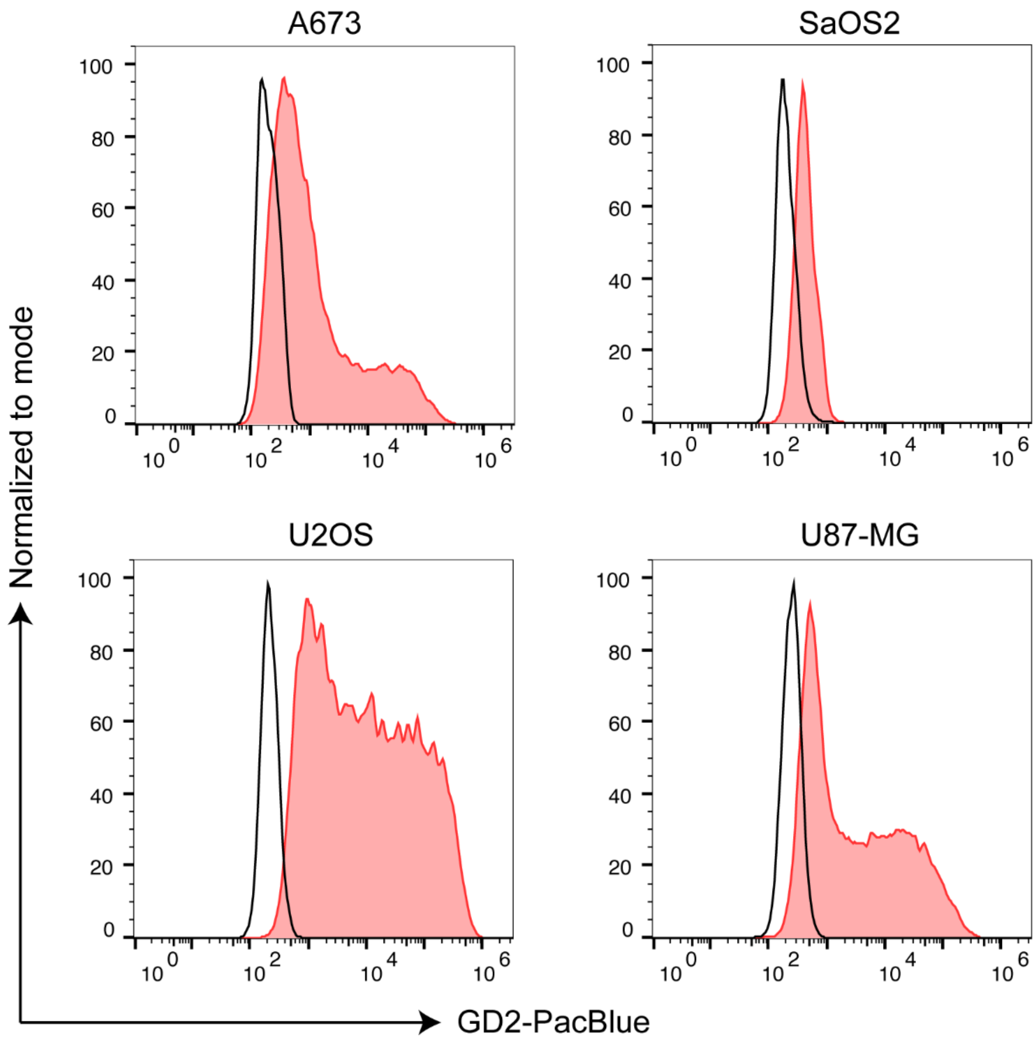

Supplemental Figure S4. Expression of GD2 in commercially available cell lines

GD2 expression was analyzed by flow cytometry. A673 is an Ewing's Sarcoma cell line. SaOS2 and U2OS are osteosarcoma cell lines. U87-MG is a glioblastoma cell line.
