# Supplementary material for: Exploiting Gangliosides for the Therapy of Ewing’s Sarcoma and H3K27M-Mutant Diffuse Midline Glioma
Source: Cancers (Basel). 2021 Jan 29;13(3):520. doi: 10.3390/cancers13030520 (PMC7866294; doi:10.3390/cancers13030520)
Supplement: Supplementary file 1 [file cancers-13-00520-s001.zip › cancers-1068769-sup/Supplemental Figure S6.pdf]

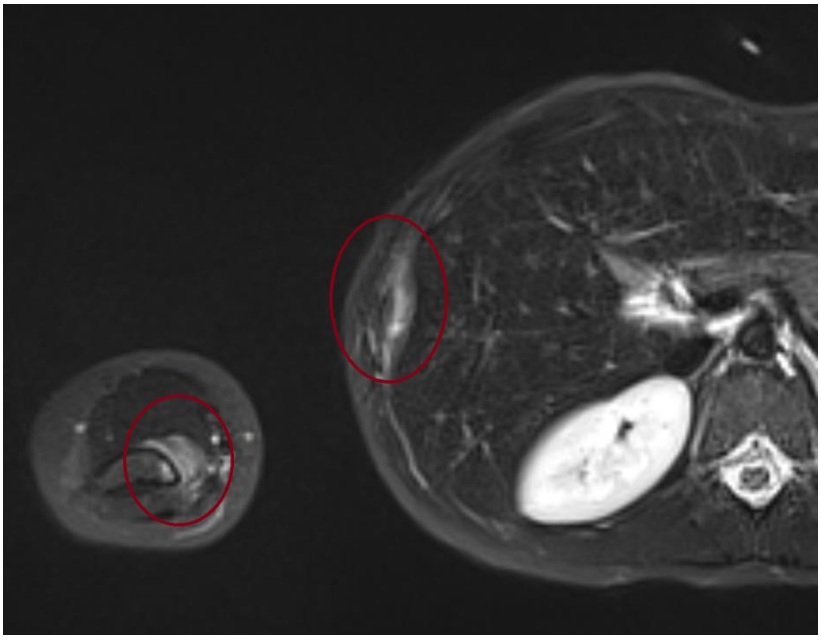

Supplemental Figure S6.

Transversal T2-weighted short-tau inversion recovery MRI sequence showing bone metastases of the right distal humerus and 9th rib at week 60. The bone metastases were detected 4 weeks after the detection of the skull metastsis that was used for the isolation of primary cells no 482 in Table 1.

.
