# Supplementary material for: Exploiting Gangliosides for the Therapy of Ewing’s Sarcoma and H3K27M-Mutant Diffuse Midline Glioma
Source: Cancers (Basel). 2021 Jan 29;13(3):520. doi: 10.3390/cancers13030520 (PMC7866294; doi:10.3390/cancers13030520)
Supplement: Supplementary file 1 [file cancers-13-00520-s001.zip › cancers-1068769-sup/Supplemental Figure S7.pdf]

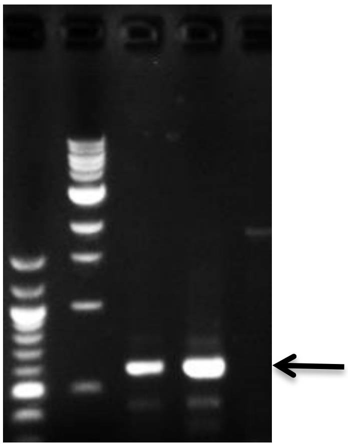

1 2

Supplemental Figure S7. Detection of the EWSR1-FLI1 fusion

RNA was extracted from primary tumor cells isolated from the tumor before (1, sample no 408) or after (2, sample no 482) the dinutuximab therapy. The EWSR1-FLI1 fusion (arrow) was amplified with specific primers and validated by Sanger Sequencing
