# Supplementary material for: Exploiting Gangliosides for the Therapy of Ewing’s Sarcoma and H3K27M-Mutant Diffuse Midline Glioma
Source: Cancers (Basel). 2021 Jan 29;13(3):520. doi: 10.3390/cancers13030520 (PMC7866294; doi:10.3390/cancers13030520)
Supplement: Supplementary file 1 [file cancers-13-00520-s001.zip › cancers-1068769-sup/Supplemental Figure S8.pdf]

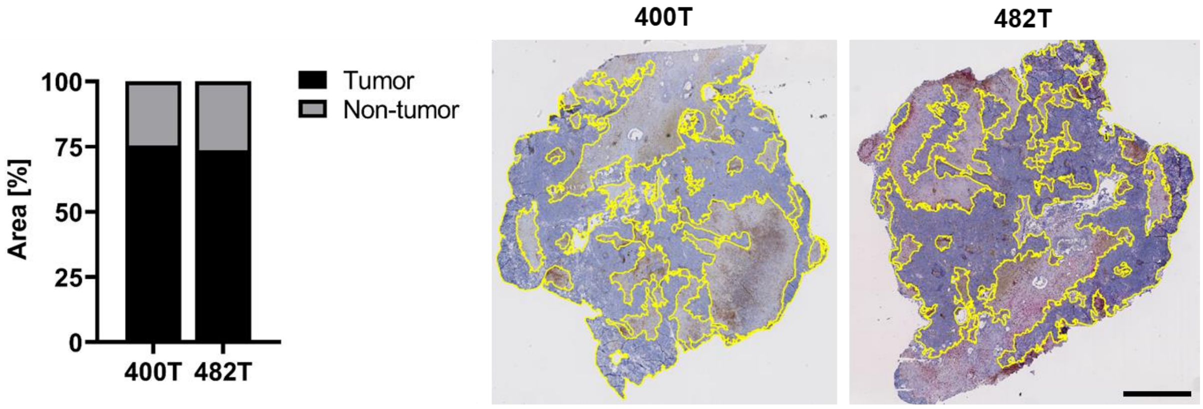

Supplemental Figure S8. Supervised machine-learning-based classification of tumor and non tumor tissue.

Irrelevant structures including background, holes and artefacts were also classified, but excluded from further analyses. Bar: 2 mm. The tumor sample 400T was isolated from a relapse in the rib (primary cells were not available for this sample) before the start of dinutuximab therapy. The tumor sample 482T was isolated from the intracranial metastasis developed under dinutuximab therapy (primary tumor cells isolated from the same metastasis have no 482 in Table 1)
