# Supplementary material for: Exploiting Gangliosides for the Therapy of Ewing’s Sarcoma and H3K27M-Mutant Diffuse Midline Glioma
Source: Cancers (Basel). 2021 Jan 29;13(3):520. doi: 10.3390/cancers13030520 (PMC7866294; doi:10.3390/cancers13030520)
Supplement: Supplementary file 1 [file cancers-13-00520-s001.zip › cancers-1068769-sup/Supplemental Figure S9.pdf]

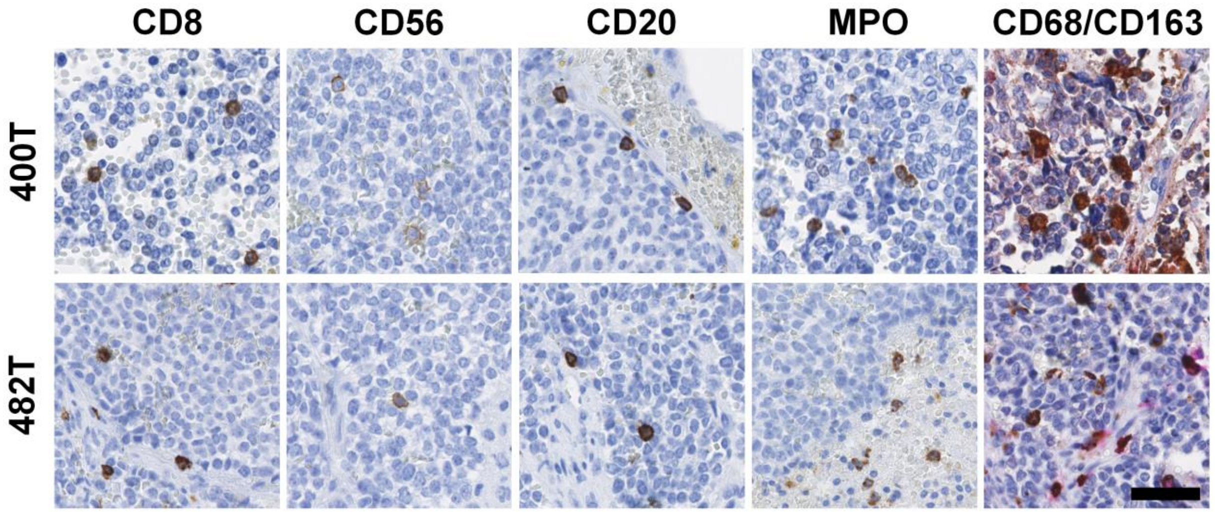

Supplemental Figure S9. Representative immunohistochemistry images

Representative immunohistochemistry images of CD8, CD56, CD20, MPO, CD68 and CD163 before (400T) and after (482T) dinutuximab treatment. The macrophage markers CD68 and CD163 were co-stained (brown/magenta). Scale bar: 50 μm.
