# Supplementary material for: Exploiting Gangliosides for the Therapy of Ewing’s Sarcoma and H3K27M-Mutant Diffuse Midline Glioma
Source: Cancers (Basel). 2021 Jan 29;13(3):520. doi: 10.3390/cancers13030520 (PMC7866294; doi:10.3390/cancers13030520)
Supplement: Supplementary file 1 [file cancers-13-00520-s001.zip › Supplemental Figure 4.pdf]

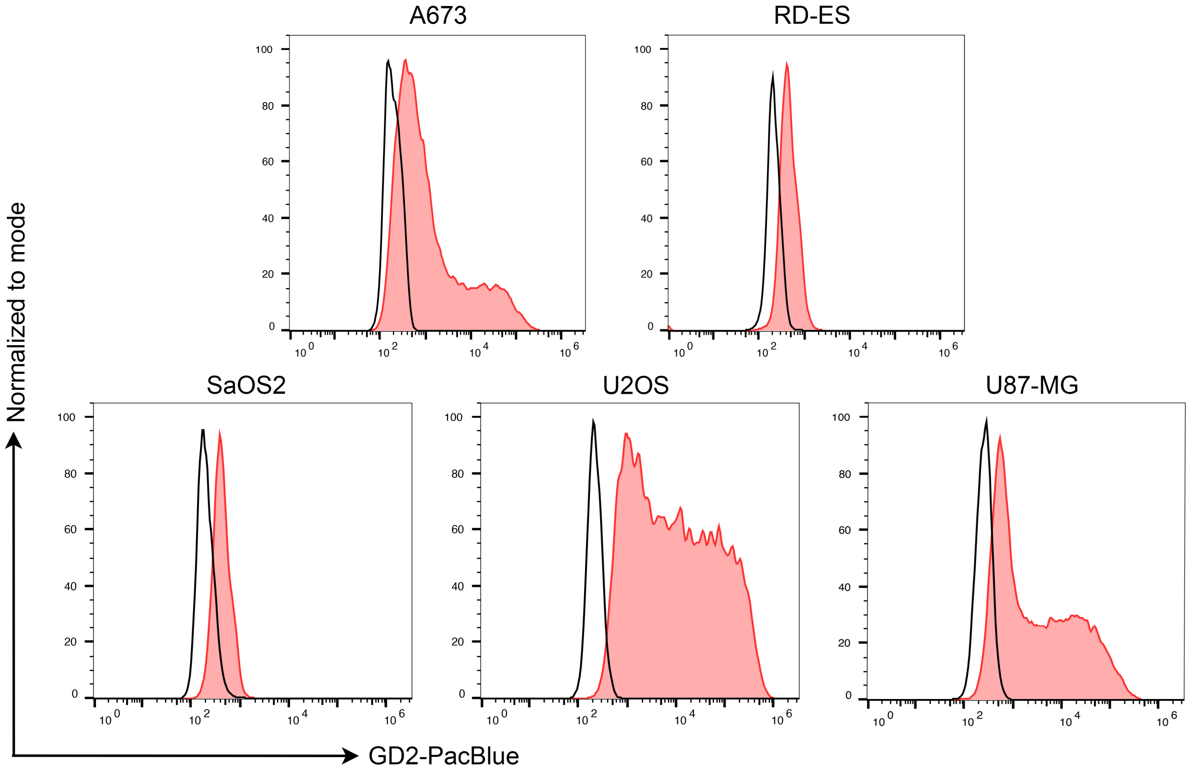

Supplemental Figure 3. Expression of GD2 in commercially available cell lines

GD2 expression was analyzed by flow cytometry. A673, RD-ES are Ewing's Sarcoma cell lines. SaOS2, U2OS are osteosarcoma cell lines. U87-MG is a glioblastoma cell line
