# Supplementary material for: Exploiting Gangliosides for the Therapy of Ewing’s Sarcoma and H3K27M-Mutant Diffuse Midline Glioma
Source: Cancers (Basel). 2021 Jan 29;13(3):520. doi: 10.3390/cancers13030520 (PMC7866294; doi:10.3390/cancers13030520)
Supplement: Supplementary file 1 [file cancers-13-00520-s001.zip › Supplemental Figure 5.pdf]

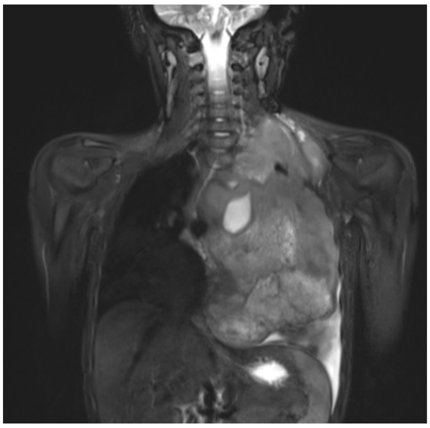

**Supplemental Figure 4. ES at diagnosis**

Coronal T2-weighted short-tau inversion recovery MRI sequence demonstrating a large mass in the left hemithorax arising from the 1st rib, which extended cranially into the supraclavicular region.
