# Supplementary material for: Exploiting Gangliosides for the Therapy of Ewing’s Sarcoma and H3K27M-Mutant Diffuse Midline Glioma
Source: Cancers (Basel). 2021 Jan 29;13(3):520. doi: 10.3390/cancers13030520 (PMC7866294; doi:10.3390/cancers13030520)
Supplement: Supplementary file 1 [file cancers-13-00520-s001.zip › Supplemental Figure 7.pdf]

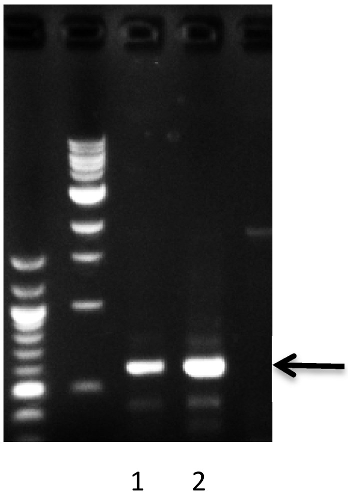

Supplemental Figure 5. Detection of the EWSR1-FL1 fusion

RNA was extracted from primary tumor cells isolated from the tumor before (1) or after (2) the dinutuximab therapy. The EWSR1-FL1 fusion was amplified with specific primers (arrow) and validated by Sanger Sequencing
