# Supplementary material for: Exploiting Gangliosides for the Therapy of Ewing’s Sarcoma and H3K27M-Mutant Diffuse Midline Glioma
Source: Cancers (Basel). 2021 Jan 29;13(3):520. doi: 10.3390/cancers13030520 (PMC7866294; doi:10.3390/cancers13030520)
Supplement: Supplementary file 1 [file cancers-13-00520-s001.zip › Supplemental Figure 8.pdf]

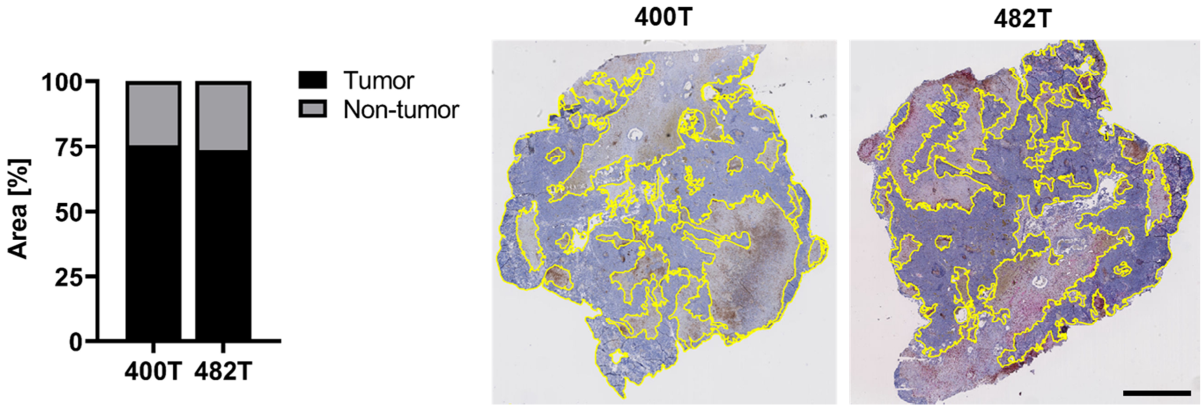

Supplemental Figure 7. Supervised machine-learning-based classification of tumor and non tumor tissue. Irrelevant structures including background, holes and artefacts were also classified, but excluded from further analyses. Bar: 2 mm.
