# Supplementary material for: Exploiting Gangliosides for the Therapy of Ewing’s Sarcoma and H3K27M-Mutant Diffuse Midline Glioma
Source: Cancers (Basel). 2021 Jan 29;13(3):520. doi: 10.3390/cancers13030520 (PMC7866294; doi:10.3390/cancers13030520)
Supplement: Supplementary file 1 [file cancers-13-00520-s001.zip › Supplemental Figure 9.pdf]

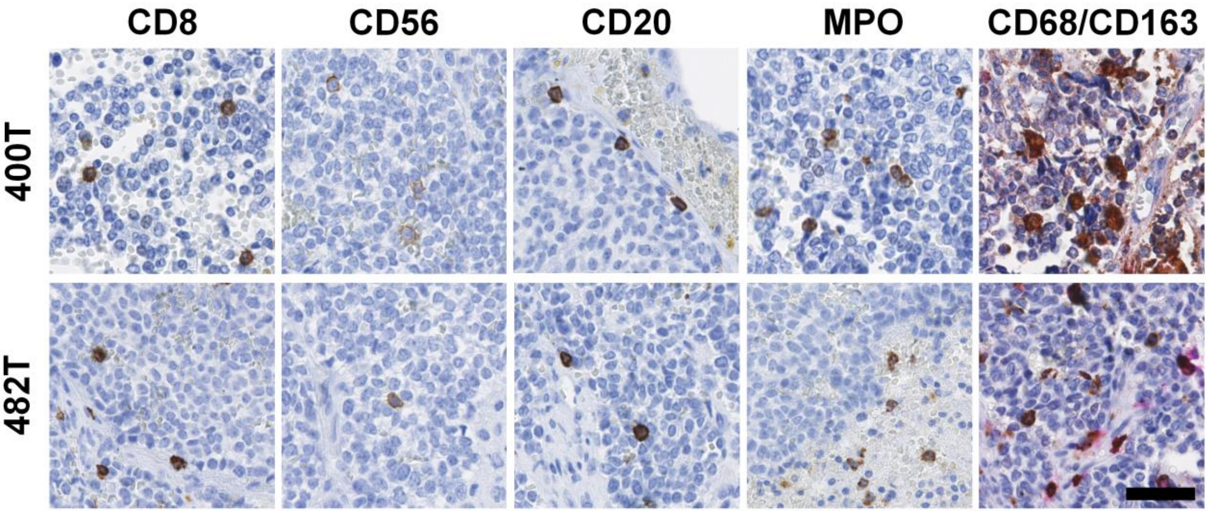

Supplemental Figure 9. Representative immunohistochemistry images of CD8, CD56, CD20, MPO, CD68 and CD163 before (400T) and after (482T) dinutuximab treatment. The macrophage markers CD68 and CD163 were co-stained (brown/magenta). Scale bar: 50 μm.
